# Biomaterials for In Situ Tissue Regeneration: A Review

**DOI:** 10.3390/biom9110750

**Published:** 2019-11-19

**Authors:** Saba Abdulghani, Geoffrey R. Mitchell

**Affiliations:** Centre for Rapid and Sustainable Product Development, Polytechnic of Leiria, 2430-080 Marinha Grande, Portugal; geoffrey.mitchell@ipleiria.pt

**Keywords:** in situ tissue engineering, biomaterials, natural polymers, synthetic polymers

## Abstract

This review focuses on a somewhat unexplored strand of regenerative medicine, that is in situ tissue engineering. In this approach manufactured scaffolds are implanted in the injured region for regeneration within the patient. The scaffold is designed to attract cells to the required volume of regeneration to subsequently proliferate, differentiate, and as a consequence develop tissue within the scaffold which in time will degrade leaving just the regenerated tissue. This review highlights the wealth of information available from studies of ex-situ tissue engineering about the selection of materials for scaffolds. It is clear that there are great opportunities for the use of additive manufacturing to prepare complex personalized scaffolds and we speculate that by building on this knowledge and technology, the development of in situ tissue engineering could rapidly increase. Ex-situ tissue engineering is handicapped by the need to develop the tissue in a bioreactor where the conditions, however optimized, may not be optimum for accelerated growth and maintenance of the cell function. We identify that in both methodologies the prospect of tissue regeneration has created much promise but delivered little outside the scope of laboratory-based experiments. We propose that the design of the scaffolds and the materials selected remain at the heart of developments in this field and there is a clear need for predictive modelling which can be used in the design and optimization of materials and scaffolds.

## 1. Introduction

Key to the survival of humans is the ability to self-heal when damaged. We are most familiar with this process when our skin self-repairs after an injury, but the same process is happening with many organs. For example, the liver is responsible for removing toxins from the blood and can be easily damaged. Therapies for liver cells create many challenges arising from the complexity of the structure of the liver and its function. The key functional or parenchymal tissue is comprised of hepatocytes which are very sensitive to the environmental architecture and when removed from the body lose both their ability to replicate and provide specific liver functions [1]. To repair the damaged liver tissue, it may be sufficient to eliminate the toxin from the diet, for example if the patient stops drinking alcohol. A developing alternative is the use of cell-based therapies and a number of successes have been reported. If the damage is too great or the toxin cannot be eliminated, then a transplant would be an option. Although such allograft transplants carry risks of rejection and other complications, the major drawback is the limited number of organs available and the suitability of the recipients. The need for alternative approaches has led to the development of the field of tissue engineering (TE). TE focuses on regenerating damaged tissues, rather than replacing them, by developing biological substitutes which restore and improve tissue function [2]. The term ‘tissue engineering’ originated at a National Science Foundation (NSF) workshop in Granlibakken, California and later published in the proceedings of the workshop by Skalak in 1988 [3]. This definition was later strengthened by Langer and Vacanti in 1993 [4] and subdivided into three main groups which are focused on (a) isolated cell implantation; (b) introduction of growth factors to the cells to stimulate cell growth and (c) the introduction of cells on or within different scaffolds which aim to mimic the extracellular matrix. The latter is the most commonly used tissue engineering approach that is associated with seeding living cells on natural or synthetic extracellular scaffolds to create an implantable substrate [5], capable of providing a functional 3D structural support system to promote the formation of a new extracellular matrix (ECM) similar to that of the damaged native tissue [6].

**Figure 1:**
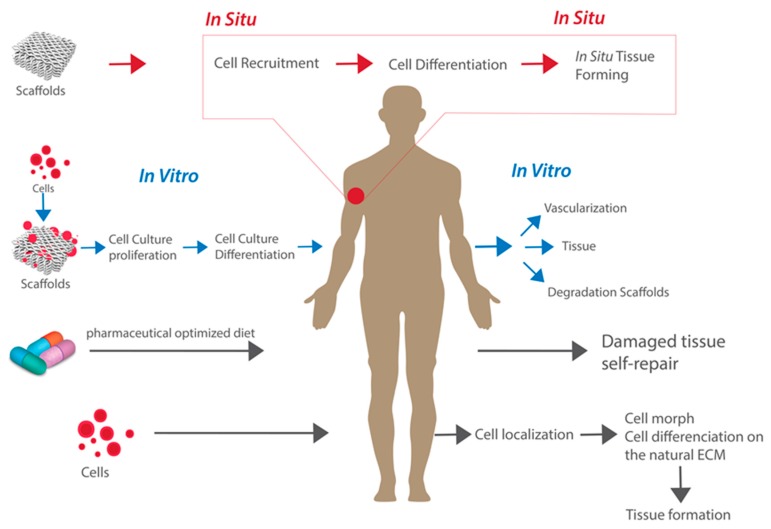
Pathways for tissue regeneration.

We illustrated these different routes to tissue regeneration in Figure 1. Each, as might be expected, has merit in different circumstances. The natural pathway is the medically favored approach in most cases but may not be possible due to the extent of the injury or diseased area. Cell-based therapies for example with respect to spinal cord injuries provide conflicting results and it is now thought that the injection of stem cells does not lead on to differentiation but rather the release of secreting factors, the paracrine effect that promotes the preservation and activity of other cells. The most widely studied tissue engineering technique employs a top-down approach in which the cells are collected, and seeded on or within a porous biodegradable scaffold, with the desired size and shape, and conditioned in an in vitro bioreactor for several weeks where it is exposed to cues including biochemical signals and mechanical stimulation which serve to guide the formation of appropriate tissue type and architecture and ensure fast matrix production [7].

The main advantage of a top-down approach is the prospect of using a variety of biomaterials and the ability to produce porous scaffolds of specific mechanical properties [8]. The use of additive manufacturing [9,10,11] has proved to be particularly effective in producing scaffolds with the required properties in a shape personalized to the patient and the injury site. However, this approach suffers some limitations such as positioning cells of multiple types with precision, achieving geometries and cell densities which are tissue specific. An additional limitation is the difficulty of the incorporation of vasculature throughout the 3D space of the tissue constructs [12]. To circumvent these limitations, ‘bottom-up’ strategies have emerged as an alternative for functional tissue engineering, where primitive building blocks composed of cells, materials, and/or bioactive factors are assembled into more complex functional units mimicking the organization and the architecture of native tissues [13]. In this approach, the construction of these primitive building blocks is achieved by different methodologies, including microscale hydrogels (microgels) which encapsulate cells [14], self-assembled cell aggregation [15,16], cell sheets [17], and 3D cell printing [18,19].

## 2. From In Vitro to In Situ

The environment required for the development of a functional tissue must mimic the native chemical cues from the organism and the biophysical cues to ensure cell survival, migration, proliferation, and differentiation [20]. Even with an appropriate scaffold, TE has a number of limitations such as the availability of a donor tissue biopsy due to restricted anatomical site access and morbidity [21]. Additionally, the tissue derived primary cells require an extensive and complicated cell expansion steps before implantation and these are often heterogeneous and as a consequence difficult to standardize making a successful translation from in vitro to in vivo difficult [19]. In addition to autologous cell source, allogeneic and xenogeneic cell sources can also be used, but they too have their own associated complications such demanding cell manipulation that involves cell isolation and expansion, immunologic responses arising from genetic differences, and the potential transmission of bacteria and viruses from the donor to the host tissue. Stem and progenitor cells can be used as an alternative route, but this is also a complicated ex vivo procedure [22]. The above mentioned limitations makes the conventional tissue engineering approach time consuming and laborious [23] and its commercialization is considered limited due to the combined difficulties of transportation and storage, making it less convenient and in particular less clinically viable [24]. The difficulties and limitations of conventional TE have given rise to a new concept known as “in situ TE” where the body’s own regenerative capability is harnessed to enable it to regenerate and heal [25]. This process is aided through the exploitation of a tissue-specific scaffold that can control the microenvironment at the injured site, that will enable the mobilization of host stem/progenitor cells to these tissues and ensures their subsequent proliferation [19]. In doing so, it eliminates the need for in vitro cell manipulation and offers an alternative therapy path that decreases the time, and resources required to successfully regenerate a tissue or organ [21,26]. It takes advantage of the natural presence of the biochemical and biophysical cues at the injured site of the living tissue by employing the living body as a more effective bioreactor that can regenerate and maintain new tissue at the site of injury in a scalable and cost-effective way [25]. More specifically, in situ TE focuses on using a target specific biomaterial constructs with or without growth factors to recruit host stem or tissue specific progenitor cells to the site of injury (Figure 1), thus allowing the regeneration of the damaged tissue without the need for cell implantation [27,28].

It is worth mentioning that although biomaterials are essential for in situ tissue regeneration, the regenerative capability of the recruited cells is of paramount importance and play a critical part in this process. This regenerative potential is known to vary with age as was clearly shown by Liu et al. [29]. In their study, they showed that in situ bone regeneration with the aid of a biomineralised scaffold was less, by means of lower mineral deposition, in elderly mice. Additionally, it is well known that different tissues regenerate at different rates due to limited blood supply as is the case of cartilage regeneration [30], thus an in situ regeneration using a biomaterial scaffold is expected to follow a similar pattern to that experienced by the native tissue but can be enhanced with the use of additives/surface treatment or specific scaffold design—which is discussed in later sections.

## 3. Biomaterials: Types and Requirements for In Situ Tissue Regeneration

During in situ tissue regeneration, a tissue-specific biomaterial scaffold, alone or in combination with growth factors, is implanted at the site of the damaged tissue. The biomaterial scaffold is expected to fully occupy the anatomy of the defect site, provide an instructive microenvironment to promote and recruit host stem cells or progenitor cells in vivo, provide stimulants to trigger native healing cascades, promote differentiation of cells and the proliferation for in situ tissue repair. The tissue also provides structural support until the new tissue is formed without the need for complicated in vitro manipulation [21]. The therapeutic outcome of using the biomaterial scaffold for in situ tissue regeneration thus depends on effective cell homing and control of cell fate [31]. Key to the methodology is the production a scaffold with the required properties tailored to the patient and the shape and volume of the damaged tissue. The properties of the biomaterial scaffold for in situ tissue regeneration may vary depending on the target damaged tissue, but generally will share some common requirements.

### 3.1. Biocompatibility and Biodegradability

Scaffold biocompatibility and biomimicry is essential for performing and maintaining essential cellular activities which include molecular level and mechanical signaling networks [11]. The incorporation of bioactive features such as biochemical stimuli, mechanical forces, and physiochemical material properties into the chosen biomaterial plays an important role in influencing cell behavior by creating an environment that stimulates a cellular response similar to that of the host tissue [32,33,34]. Equally important is the biodegradability of a biomaterial, with non-cytotoxic degradation by-products and minimal fibrosis and associated foreign body reaction. The degradation rate is vital for successful in situ tissue regeneration. In this respect, the biomaterial can be designed to provide a dynamic control of the porosity of the scaffold, as well as its mechanical integrity and bioactivity [26]. It has been shown that the degradability rate can influence the mechanical behavior of the scaffold, thus a rapid degradation could lead to early mechanical failure, such as the case of bone tissue regeneration where the scaffold usually endures mechanical loading during the regeneration process. On the other hand, if the degradation process is too slow, complications related to inflammation could also take place [35].

Degradation can occur globally by hydrolysis such as in the case of dextran and poly(lactide-co-glycolide) (PLGA) or by enzymes [36]. Mann et al. showed that photopolymerizable polyethylene glycol (PEG)-based hydrogels are able to be tailored to respond to in situ tissue remodeling by being chemically modified to be degraded by collagenase and elastase, thus permitting cell migration and cell proliferation [37].

### 3.2. Surface Topography and Chemistry

The scaffolds must possess suitable surface topography and chemistry for the promotion of cell seeding, attachment, and proliferation [27]. It has been shown that surface characteristics play an important role in the extent of cell adhesion and cellular spreading on the surface of biomaterial scaffold [38]. In addition to the type of biomaterial used to fabricate the scaffold, its surface treatment or inclusion of nanoparticles may in fact affect the surface roughness and results in improved scaffold bioactivity [39]. Martins et al. showed that the incorporation of calcium phosphate coatings as hydroxyapatite particles or whiskers, led to an improved bone formation in porous biomaterials [40]. Yavari and co-workers demonstrated that applying a surface treatment to bone scaffolds leads to improved bone mineralization through alterations to the scaffold surface chemistry and roughness [41]. Another interesting example is vascular tissue engineering where it is essential to apply a surface treatment to the fabricated vascular graft as it is considered vital to avoid thrombus formation [42].

### 3.3. Scaffold Architecture

The scaffolds must have an appropriate architecture to facilitate the flow of nutrients and the formation of capillary blood vessels through infilling, thus regulating cell growth and infiltration as well as recruitment to the injury location. In addition, the structure of the scaffold can also be used to encapsulate bioactive molecules such as growth factors, genes, and cytokines [21], which are considered crucial for controlling the in vivo microenvironment and thus optimizing the in situ tissue regeneration process by promoting cell-specific recruitment and growth [43,44].

Thevenot et al. have studied the incorporation of biomolecules into biomaterials to enhance cell recruitment. In one study, PLGA scaffolds incorporated with stromal cell-derived factor 1 (SDF-1α—a potent factor for stem cells recruitment) were found to recruit a greater number of stem cells, in order to increase new blood vessel formation, decrease fibrotic response, as well as regulate the inflammatory cell responses compared with pure PLGA scaffolds [45].

**Figure 2:**
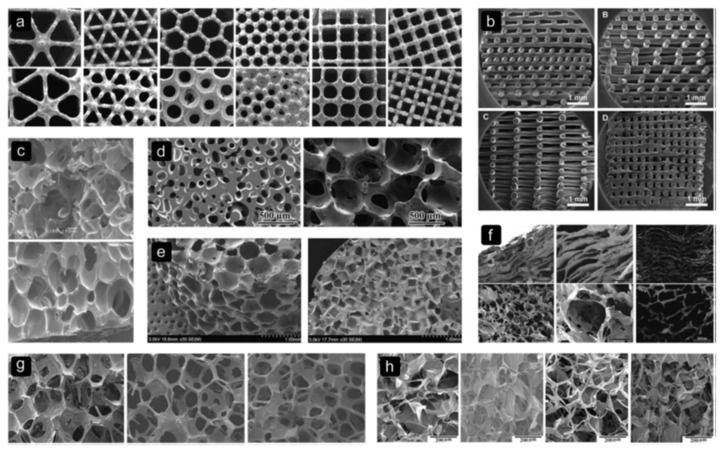
Examples of different pore sizes, shapes, and biomaterials for scaffolds for tissue engineering. (**a**) Titanium (Ti6Al4V), (**b**) Starch poly(ε-caprolactone) (SPCL), (**c**) poly(lactide-co-glycolide) (PLGA), (**d**) Bioactive glass (BG), (**e**) poly(propylene fumarate) (PPF), (**f**) collagen-apatite, (**g**) Mesoporous bioactive glass (MBG), and (**h**) Silk fibroin (SF). Reproduced from [39], CC BY 3.0 license.

Cell seeding, behavior, and response are also found to be significantly influenced by the architectural properties of the biomaterial scaffold. It has been shown that the scaffolds used in bone tissue regeneration must possess optimum pore size, pore shape, porosity, interconnectivity, and fiber-orientation [38,39]. In a wide-ranging review by Bobbert et al. [39] on the architecture of bone substitute materials, it was shown that scaffolds with small pores (200–300 µm) present a better environment for the seeding of cells, however, limit cell sustainability, proliferation, and differentiation. Additionally, it was shown that scaffolds which adopt “ladder-like”, “rectangular pores”, and those with “large spherical pores” are found to be more inclined to fail than those with uniform small and rounded pore structures [5]. For example, Ti6Al4V structures (Figure 2a) fabricated with different shapes of pores exhibited a variation in the mechanical properties and fatigue strength for the range of unit cells with similar porosity (Figure 2). Bobbert et al. [39] concluded that designing what they referred to as a ”tortuous void network” would in fact help control the passage of the cell suspension through the scaffold, thus increasing the surface area and available time for cell attachment.

**Figure 3:**
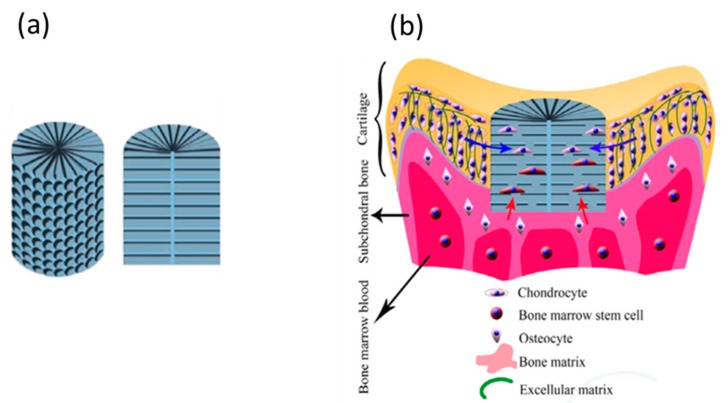
Schematic illustration to show (**a**) the radially oriented pores scaffold, O-PLGA and (**b**) its implantation in an osteochondral defect in a rabbit model. The blue and red arrows in (**b**) refer to the bioactive interflow from neighbor cartilage and subchondral bone layers, respectively. PLGA—poly(lactide-co-glycolide). With permission [48].

**Figure 4:**
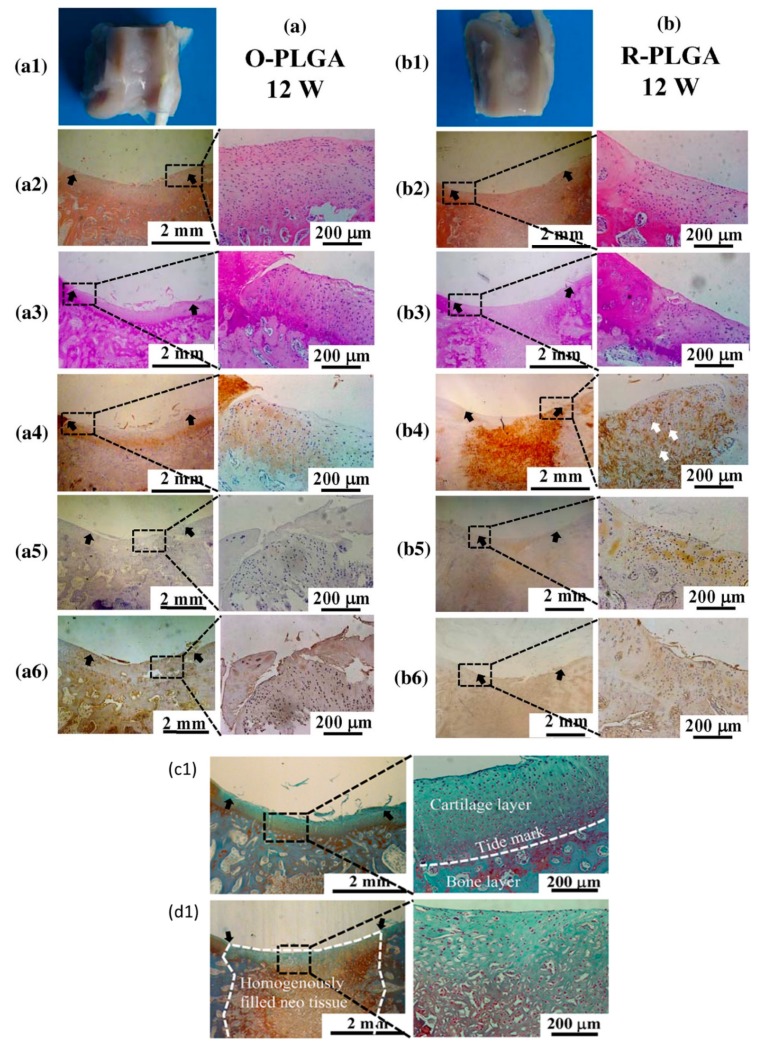
Assessment of the osteochondral defects regeneration using (**a**) O-PLGA and (**b**) R-PLGA for 12 weeks, respectively. (**a1,b1**) Gross view of the scaffold in the osteochondral bone, (**a2,b2**) hematoxylin and eosin staining images, and (**b3,b3**) periodic acid Schiff staining of glycosaminoglycans. Immunohistochemical staining of collagen Type II (**a4,b4**), collagen Type I (**a5,b5**), and collagen Type X (**a6,b6**), respectively. The black arrows in the images are used to indicate the edges of the defects, while the white arrows highlight debris of in the PLGA scaffold. Safranine O and fast green staining of glycosaminoglycans (red) and collagen (green) of O-PLGA (**c1**) and R-PLGA (**d1**) respectively. Reproduced with permission from [48].

Sussman et al. [46] demonstrated that when porous biomaterials with an optimum pore size were subcutaneously implanted in mice, the optimum pore size can control the spatial organization of the M1 (pro-inflammatory) or the M2 (pro-healing) macrophage response. Thus, in situ TE can be used as a powerful tool to control inflammatory response, which significantly impacts tissue regeneration in vivo [47]. Dai et al. [48] investigated the use of poly(lactide-co-glycolide), PLGA scaffold with a radially oriented pore architecture, O-PLGA (Figure 3), in an in situ regeneration of osteochondral defects in rabbits. Their results revealed that the O-PLGA scaffold design was shown to enable cell infiltration and bioactive interflow between neo-host tissues more than a randomly porous R-PLGA scaffold in vivo. These results succeeded in emphasizing the significance of using structured scaffolds to optimize the regeneration of native tissues in vivo. Their histological analysis results demonstrated the clear formation of tide mark in the O-PLGA [Figure 4(a3)] in comparison to the R-PLGA [Figure 4(b3)] and a significant presence of chondrocytes regularly spread in the cartilage layer [Figure 4(a4)]. Furthermore, an observable boundary was seen between the two layers, signifying the simultaneous regeneration of both the cartilage and the subchondral bone layers, which is not observed with the R-PLGA scaffold [Figure 4(c1,d1)].

Arora et al. [49] also showed that scaffolds with oriented pores are considered more suitable and efficient for cell growth, nutrients and waste exchange, and regular deposition of ECMs. Additionally, such architecture offers the ideal setting for the critical cell–cell, cell–matrix, and neo-host tissue interaction, and improves the mechanical properties in comparison to randomly porous scaffolds. In general, the tendency towards oriented-porous scaffolds stems from the need to mimic natural tissues such as tendons, cartilage, bone tissue, nerves, etc., all of which naturally possess an oriented structure that helps enhance their biological and mechanical functions [50], hence oriented-structures are more favorable when designing a scaffold for in situ tissue regeneration.

### 3.4. Mechanical Properties

The scaffold must have equivalent mechanical properties to the native tissue in order to offer effective support during the regeneration process [28]. It has been shown that the modulus of the biomaterial significantly influences cell adhesion, distribution, proliferation, and differentiation as was demonstrated by the work on highly elastic poly-L-lactic acid (PLLA) constructs where chondrogenic differentiation was significantly improved in comparison to the rigid Poly(lactic acid) (PLA) and PLGA ones [51]. Bhumiratana et al. [52] incorporated hydroxyapatite in silk sponges in an effort to fabricate a composite scaffold that stimulates the formation of mineralized bone tissue by human mesenchymal stem cells (hMSC). Their results showed enhanced mechanical properties by two mechanisms, one was attributed to the osteoconductive nature of the hydroxyapatite and the second to the fact that it provided nucleation sites for the mineral phase. Additionally, in the case of bone defects, it is reported that the scaffold stiffness has an influence on its integration into the local native tissue especially when there is a significant difference in the stiffness. For example, scaffolds composed of polymers would certainly have a stiffness that is different to those composed of metal or ceramics and as a result of this mechanical difference, stress and deformation profiles would also be different throughout the scaffold [53,54].

### 3.5. Handling Properties

Biomaterials utilized for in situ tissue regeneration can be designed to be adaptable so as to better serve the necessities of the damaged native tissue such as scaffolds, hydrogels, membranes, tubes, micro and nano spheres [55,56,57,58,59], and use manufacturing methods, such as additive manufacturing, that are cost-effective [10,11]. From a surgical perspective, it is desirable for the biomaterial to be easily manipulated into different forms and sizes to allow in situ tissue regeneration tailored to the individual patient and their injuries as in the case of bone defect treatments [35,60]. When hydrogels are used for in situ tissue regeneration it is important that they display optimum injectability properties relevant to the surgical procedure as was highlighted in a recent study by Steele et al. [61]. In this study, they evaluated in vivo biocompatibility and hemocompatibility using an animal model where they performed subcutaneous and intramuscular injections of hyaluronic acid–nanoparticle (HA–NP) hydrogels and compared the results to a PBS control. Their findings revealed no significant difference in immunological response or the presence of fibrosis. They also examined the hemocompatibility of the hydrogel for cardiovascular applications, including intramyocardial delivery, and reported that intracardiac injection of HA–NP hydrogel in rats resulted in insignificant gross or histological changes in comparison to PBS injected-rats. It may well be that the recent developments in 4D printing are able to contribute to the design of scaffolds to deliver therapies without major surgical intervention [62].

There are three classes of biomaterials used in in situ tissue regeneration: polymers (natural and synthetic), bioceramics, and ECM-based biomaterials.

## 4. Biomaterials

### 4.1. Synthetic Polymers

Synthetic biomaterials for use in in situ tissue regeneration are based on degradable synthetic polymers and are fabricated using various assembly approaches in order to produce structures with enhanced physical and mechanical properties such as stiffness, degradation, and porosity [63]. One of the advantages of using synthetic biomaterials is the ability to produce patient-specific scaffolds to match the target anatomy as well as fit the required physical and chemical properties of the injured tissue [64]. In this way, synthetic polymers have no immunological concerns [65] and a higher degree of processing flexibility, thus it is easier to program their biodegradation rate, mechanical properties and microstructure [66]. Talacua and co-workers investigated the use of resorbable poly(ε-caprolactone) (PCL) grafts as capillary blood vessels, which are filled with a fibrin gel and the monocyte chemoattractant protein 1, in a rat model of abdominal aortas. After 3 months in situ, their study revealed higher structured systems with a layer on the inside formed from elastin fibres coated with endothelium cells, as well as a layer of smooth muscle cells at the centre of the structure [67]. Moreover, synthetic biomaterials can be designed to have functional sites by coupling with biomolecules [68] and there is less biosafety concerns in particular with regards to the host tissue response and disease transfer in comparison to natural polymers [69]. Of the interesting groups of biodegradable synthetic polymers used in tissue engineering are aliphatic polyesters such as PLA, PCL, polyglycolide (PGA), and their copolymer PLGA [70]. These synthetic polymers undergo degradation, involving the hydrolysis of the ester groups in their backbone chains, into non-cytotoxic products [71]. More importantly, the properties of this class of biomaterials can be tailored by selecting the molecular weight distribution, the nature of the porous architecture, and the selection of the degradation rate for a specific tissue regeneration application by variation of the monomer composition of copolymers [72]. These factors amongst others will determine the resultant mechanical properties. Two important polymer chemistry tools in this respect are the use of copolymerization and blending to obtain tailored degradation rates. These polymers are among the few synthetic polymers already approved by the US Food and Drug Administration (FDA) for clinical applications such as surgical sutures and some implantable devices [68]. One of the most common synthetic biomaterials is PLGA where it is extensively used in bone and cartilage tissue regeneration [73]. However, conventional synthetic polymers have disadvantages due to the lack of functional groups resulting in their reduced capability to combine with bioactive moieties to strengthen their cell affinity [12]. Functional synthetic polymers have gained popularity due to their facile design and modification which enables them to provide bioactive signals to enhance the spatiotemporal cell-biomaterial interaction [74]. Other examples of synthetic biomaterials and their applications are shown in Table 1.

Synthetic polymers enable scaffolds to be manufactured with precision and with controlled architecture and target mechanical properties. There are a limited number of polymers which are FDA approved for clinical applications.

### 4.2. Natural Polymers

Natural polymers, derived from polysaccharides and proteins, are types of biomaterials that have excellent biodegradable and biocompatible properties as well as other features mimicking the extracellular matrix (ECM) making them a very attractive choice for TE applications [31]. Popular polysaccharide-based natural polymers that have been widely used for in situ tissue regeneration include fibrin, alginate, hyaluronic acid and chitosan [82]. Chitosan, which is derived from chitin, is the second most abundant biosynthesized biomaterial [83]. Chitosan exhibits a cationic nature which makes it an ideal system for the delivery of anionic glycosaminoglycans, growth factors, cytokines, and genes [84]. Another example is hyaluronic acid (HA), which is found in the extracellular matrix of many tissues, is widely used in laboratory studies of cartilage repair due to its immune and biological responses including proliferation, morphogenesis, and wound repair [85]. It can be easily modified to form hydrogels that are known to promote better cartilage regeneration than hydrogels based on polyethyleneglycol PEG hydrogels [86] and used to deliver growth factors such as VEGF and bone morphologic protein (BMP) [87,88].

Proteins, such as collagen, fibrin, gelatin and silk, form the other class of natural-based polymers [89,90]. Collagen, the most abundant type of proteins in humans, is an FDA-approved biomaterial due to its ease of processing and minimal inflammatory and immune responses [20], thus frequently used in biomedical applications such as wound dressings and artificial skin [91]. Shi and co-workers [92] demonstrated that the conjugation of collagen scaffolds with antibodies specific to stem cells could capture stem cells at a wound site and promote cardiomyocyte regeneration in a mouse model. Other examples of protein-based natural polymers are shown in Table 2.

More recently, Wu and co-workers fabricated an electrospun silk fibroin scaffold and used Polydopamine (PDA) to modify the surfaces of the scaffolds which were further modified using E7, a newly discovered peptide with specific affinity for bone marrow mesenchymal stem cells (BMSCs) in a rat calvarial bone defect model [93]. Their findings showed that these enhanced electrospun scaffolds had led to improved hydrophilicity, enabling cell proliferation and adhesion, and furthermore boosted the osteogenic differentiation of BMSCs by creating favorable osteoinduction conditions under the synergistic effects of PDA and E7 both in vitro and in vivo.

However, there are some shortcomings to the use of natural biomaterials such as relatively poor mechanical properties limiting their applications in anatomical sites with demanding mechanical loading such as hard tissue regeneration [94]. Thus, in order to improve such properties, natural polymers are often combined with synthetic ones to produce hybrid biomaterials that enjoy the advantages of both classes of polymers without compromise (Table 3).

Natural polymers offer the advantage that they may come closer to mimicking the ECM, but suffer from the difficulty of processing the material into the required shapes whilst maintaining the biological function.

### 4.3. ECM

ECM-based biomaterials are derived from decellularized ECM (dECM) tissues and these have been extensively explored for in situ tissue regeneration due to the fact that they provide an environment that is very similar to that of the native tissue ECM especially where cellular behavior is concerned [118]. Decellularized extracellular matrix is naturally the closest scaffold to nature, retaining its unique micro- and macro-structural design features as well as its complex composition [119]. The decellularization process preserves the structural integrity and thus ensures an intact vasculature [120].

Decellularized urinary bladder matrix and small intestinal submucosa are used as a source tissue for ECM-based biomaterials [121]. They are reported to maintain the necessary bioactive features to stimulate positive remodeling effects when implanted in vivo. These bioactivities include recruitment/differentiation of endogenous stem/progenitor cells and the modulation of the host immune response. One example was demonstrated with a study conducted by Dziki and co-workers [122] in patients (*n* = 13) who were treated for a volumetric muscle loss with porcine-derived ECM sheets harvested from either the urinary bladder, the small intestine, or the dermis. These sheets of ECM materials were placed in contact with the healthy tissue for subsequent in situ remodeling. Six months after implantation, and regardless of the type of ECM employed, their findings demonstrated that patients improved their strength by an average of ~37% and showed ~27% improvement in range-of-motion tasks in comparison with pre-operative performance. The results further showed that these ECM-sheets promoted the local recruitment of perivascular stem cells with the downstream formation of new functional skeletal muscle. More examples are shown in Table 4. The main disadvantage of dECM is donor shortage. However, ECM-based biomaterials can also be derived from in vitro cultured cells [123] and these have already been successfully used clinically for bone and cartilage regeneration [124]. In another study, Lih et al. fabricated biomimetic PLGA/ECM of decellularized porcine kidney scaffolds for kidney tissue regeneration in a mouse model [125]. Their results showed an increased number of kidney-related cells in the PLGA/ECM group as compared to the PLGA control, and the scaffold degradation rate was also found to be faster in PLGA/ECM scaffolds than in the control PLGA scaffolds. They further demonstrated that ECM-derived bioactive molecules in PLGA/ECM scaffolds have encouraged cell growth and metabolic acids. The hydrolytic degradation of PLGA and the enzymatic degradation of the ECM were both accelerated through the actions of enzymes secreted by active living cells. Therefore, in comparison to the control PLGA scaffold, the rapidly biodegradable PLGA/ECM scaffold is thought to be a more favorable microenvironment for cell proliferation, migration, and recruitment.

Some of the reported problems identified with the use of decellularized tissue scaffolds is the accelerated degeneration with lack of cell repopulation and remodeling evidence [126]. Recently, Dai et al. fabricated a scaffold of a porous matrix metalloproteinase degradable poly (ethylene glycol) hydrogel and decellularized porcine aortic valve in a rat subdermal model [127]. The hydrogel was loaded with stromal cell-derived factor-1α (SDF-1α) which, consequently, served to turn the decellularized scaffold biologically active. Such bioactivity caused by the SDF-1 α is thought to stimulate the in vivo tissue recellularization, by attracting progenitor cells from the bloodstream, modulating immune responses and being pivotal in tissue repair, remodeling and regeneration [128]. Another study also demonstrated that SDF-1α could control valve cell phenotype and is involved in scaffold recellularization and remodeling by stimulating the attraction of stem cells [129]. Dai et al. results demonstrated that their hydrogel surface layers provided a niche for cell activities and helped protect the decellularized scaffold from rapid degeneration, inflammation, and calcification resulting in an improved recellularization and remodeling processes of the implanted decellularized heart valves [127].

## 5. Bioceramics

Bioceramics are a class of ceramic materials specifically used for the repair and reconstruction of damaged tissues of the body. Bioceramics are categorized into the following groups: nearly inert (based on alumina and zirconia), bioactive (based on bioactive glass), and resorbable ceramics (based on β- and α-tricalcium phosphate [136]. The most commonly employed bioceramics for in situ TE are those used for bone tissue regeneration such as hydroxyapatite (HAp), calcium phosphates (CP), and tri-calcium phosphate (TCP) with different calcium to phosphate ratio and crystallographic structure, which in turn influences the degree of their solubility [137]. Such bioceramics clearly demonstrate good biocompatibility, bioactivity, osteoconductivity [12], and osteoinductivity that are essential for the initiation of in situ bone regeneration even without the use of inductive factors [138]. Additionally, it has been reported that Ha and TCP present no immunogenicity or toxic side effects [139].

**Figure 5:**
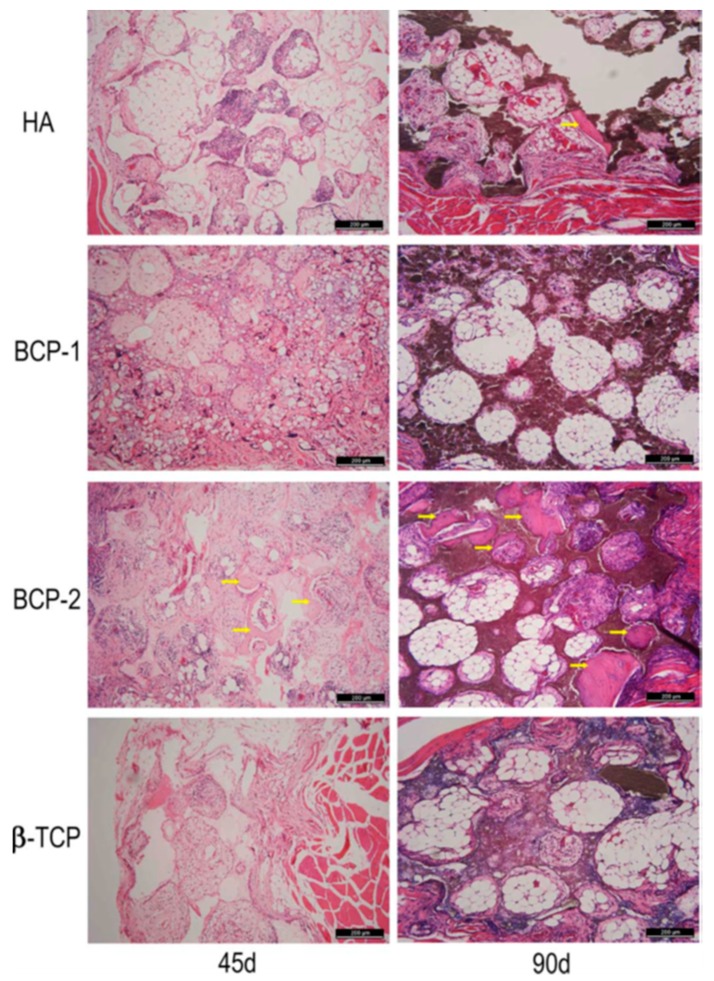
Photomicrographs of hematoxylin and eosin staining for the decalcified sections of porous Calcium Phosphate-based ceramics implanted into the thigh muscles of mice for 45 and 90 days. Yellow markers indicate the newly formed bone. Bar, 200 µm, reproduced with permission from [148].

In bone tissue regeneration, Samavedi et al. showed that certain CP ceramics are osteoinductive and that their osteoinduction property depends on the surface chemistry, which can consequently influence protein adsorption and promote cell differentiation through cell–ECM interactions [140]. However, present CP ceramics still face many challenges to meet the requirements of regenerative medicine, which include the relatively low bioactivity of the ceramic in comparison to the natural bone tissue, degradation kinetics which do not match new bone formation, and exhibiting poor machining performance to process them into specific shapes [141]. Additionally, a critical problem which restricts their wide clinical application is their poor mechanical property and brittleness. Their use to date is limited to either non-loadbearing implants, such as HAPEX as a middle ear implant [142] and bone defect filling material in the oral cavity and skeleton, or as coating on dental and orthopedic metallic implants [143]. This mechanical inferiority can be overcome by creating polymer and ceramic composites in a variety of forms such as nanofibers, foams, hydrogels, and 3D printed scaffolds for tissue-specific regeneration [144,145]. The combination of polymers and ceramics not only improves the mechanical property of the final construct but also offers biological benefits as was demonstrated by Meka et al. who showed that the inclusion of bioactive ceramic nanoparticles in the PCL composite scaffolds could possibly substitute the use of expensive bone stimulating growth factors [146]. Song and co-workers implanted biphasic calcium phosphate (BCP) granules in the dorsal muscle of lumbar region in a canine model to demonstrate how bone-marrow-derived mesenchymal cells (BMSCs) migrated from bone marrow to where new bone formation was induced without the introduction of growth factors [147]. In another study, Wang and co-workers [148] investigated the effect of chemical composition on protein (BMP-2) adsorption and osteoinductive potential in four types of bioceramics: hydroxyapatite (HAp), β-TCP, biphasic calcium phosphate-1(BCP-1), and biphasic calcium phosphate-2(BCP-2) (the ratios of HAp to β-TCP in BCP-1 and BCP-2 were about 70%/30% and 30%/70%) in a Balb/c mouse model. Histological and histomorphometric analyses of their results revealed that porous BCP-2 demonstrated a stronger osteoinductive capability than the other three groups of ceramics. The highest expression of BMP-2 and osteocalcin (OCN) was also achieved in the BCP-2 group. The amount of BMP-2 present in the local microenvironment of the implant is thought to influence the osteoinduction of porous CaP ceramics (Figure 5). Recently, Li et al. fabricated nanocrystalline of biphasic Calcium Phosphate (BCP-N) granules by combining alginate gelatinization with microwave hybrid sintering methods, and investigated the in vivo osteogenesis potential of the resultant materials in a rabbit mandible critical-size bone defect [149]. Their results showed that the nanotopography in BCP-N might be responsible for the stronger osteoinductivity and bone regenerative ability than exhibited by microcrystalline biphasic CP (BCP-G) and a commercially available type (BAM^®^, National Engineering Research Center for Biomaterials of Sichuan University, China).

Resorbable ceramics such as β- and α-TCP have the advantage of degrading gradually over a period of time while the natural host tissue regenerates itself and replaces the degrading scaffold [150]. On the other hand, hydroxyapatite (HAp) possess a porosity similar to that of native bone tissue thus promoting bone growth within the pores of the scaffold [151]. Macroporous HAp scaffolds with interconnected oval shaped pores have been found to exhibit enhanced cellular functionality and supports osteoclast differentiation in comparison to the results found for dense HAp scaffolds [152]. Additionally, it has been reported that during the degradation process of HAp, calcium and phosphate ions are released which help to induce osteogenic response and contribute to the osteoinductivity of the HAp scaffold [153]. However, its use as a scaffold material is limited because of its low mechanical properties and particularly slow rate of resorption [154]. Table 5 shows further examples of bioceramics.

Bioceramics bring a substantial history of applications in medicine and it has been clearly demonstrated that it is possible to generate scaffolds with precise complex macro-architectures. Clearly in some areas such as in bone regeneration they offer advantages of similarity in terms of chemical composition and some of the key properties of bone such as conductivity and surface topography.

The choice of material for a scaffold is largely determined by the requirement of tissue equivalent. Bioceramics can present a valuable option in the case of bone regeneration with the potential for producing a hierarchal structure at the macro, micro, and nano-levels, thus mimicking the native tissue and ensuring osteoinductivity. However, an extension of this to other tissue types is clearly inappropriate. By combining bioceramics with polymers there is a greater flexibility to achieve tissue equivalence as well as improving the overall mechanical properties. This approach has been explored by several researchers [160].

## 6. Future Prospects

In situ tissue engineering extends the family of therapies available for regenerative medicine in the field of regenerative medicine. The in situ approach avoids the necessity to remove cells from a patient and to place them in environments such as a bioreactor where the physiological environment may lead to a loss of function or the delivery of inappropriate signals. An often stated problem with the ex-situ TE process is the long delays between cell sampling and implantation especially in, for example, coronary interventions with the need for the development of small vascular grafts [161].

This review has shown that much of what has been learnt in terms of biomaterials for the development of scaffold-based ex-situ tissue engineering can in part be transferred to in situ tissue engineering and so the development of new therapies may be expedited. It is also the case that new developments in additive manufacturing will be particularly helpful in the development of realistic scaffolds for use with in situ tissue engineering which reflect the actual complexity of the extracellular matrix and the environments required for regeneration. One of the emerging developments in additive manufacturing is 4D printing [62], in which a 3D object is manufactured with a specific form and using an external stimulus, such as temperature, pH, electric and magnetic fields, which is used to initiate a transformation of the material such that the form of the object changes to an alternative design. We can foresee such materials and concepts of particular value in the design of therapies which do not need major surgical intervention to deliver in situ tissue engineering.

In all scaffold-based tissue engineering, much is made of degradable or resorbable materials especially polymers, and although such concepts are central to regenerative medicine, there appears to be little work report on how to design such materials of scaffolds to meet the personalized needs of specific patients and injuries. We see that much work is focused on biopolymers and other natural materials and although these may have advantages in biocompatibility and with degradation, they present many challenges in precisely shaping the scaffold to the particular site of the required therapy and its personalization to the patient. The hybrid nature of bone means that bioceramics present interesting opportunities linked to the need for mineralization and the generation of a structure which mimics bone. This is particular the case for bone generation with therapies to cope with bone disease, especially osteoporosis.

Most publications in this area highlight the possibility of tailoring the properties of the scaffolds to match the tissue environment but there appears to be limited work which is focused on predictive modelling which can be used to support material innovation and scaffold design.

As a number of authors identify, regeneration medicine has created much promise but delivered little outside the scope of laboratory-based experiments (EUROPEAN INITIATIVE FOR REGENERATIVE MEDICINE. Final report 2016 A Webster: https://ec.europa.eu/futurium/en/content/european-initiative-regenerative-medicine downloaded September 2019). The development of in situ tissue engineering therapies which do not require significant surgical intervention using 4d printed scaffolds may provide the much needed stimulus to this field.

## 7. Conclusions

In situ tissue engineering extends the family of therapies available for regenerative medicine in the field of bone regeneration. Much of the understanding of scaffolds and scaffold materials on scaffold-based ex situ tissue engineering may be in part transferred to the in situ methodology, perhaps enabling a faster development cycle. New developments in 4d printing provide specific opportunities for the development of therapies without major surgical intervention. The design of the scaffolds and the materials selected remain at the heart of developments in this field and there is a clear need for predictive modelling which can be used to optimize the design of materials and scaffolds.

## Figures and Tables

**Table 1:** Some examples of various synthetic polymer-biomaterials used for in situ tissue regeneration.

Application Tissue	Biomaterial *	Animal Model	Reference
Bone	PLA	Rat calvarial bone defect	Gómez et al. [75]
	P(HEMA)	Rabbit femoral defect	Mabilleau et al. [76]
	PEUU	Rat myocardial infraction model	Fujimoto et al. [77]
Cartilage	PGA	Sheep cartilage defect	Erggelet et al. [78]
	PLGA	Rabbit articular osteochondral defect	Dai et al. [48]
Heart Valve	Polycarbonate bis-urea (PC-BU)	Sheep pulmonary valve	Kluin et al. [23]
	Polyester-urethane	Sheep aortic valve	Yosuke et al. [79]
Periodontal Tissue	PLGA	Canine periodontal	Herberg et al. [80]
Blood Vessels	PLLA/PCL	Rat abdominal aorta	Jiang et al. [81]

* PLGA—poly(lactide-co-glycolide), PEUU—polyester urethane urea, PCL—poly(ε-caprolactone), PGA—poly(glycolic acid), PLA—poly(lactic acid), P(HEMA)—poly(2-hydroxyethyl methacrylate), PLLA—poly(l-lactic acid).

**Table 2:** Recent examples of natural polymer biomaterials used for in situ tissue regeneration.

Application Tissue	Biomaterials	Animal Model	Reference
Bone	Silk fibroin	Rat critical size calvarial bone defect	Wu et al. [93]
	Chitosan/silk-Fibrin	Rat calvarial bone defect model	Wu et al. [95]
	Fibrin	Rat cranial defect	Woodruff et al. [96]
	Gelatin	Mouse maxillae	Kodama et al. [97]
	Gelatin	Rat calvarial bone defect model	Feng et al. [98]
Kidney	Collagen	Renal ischemia/reperfusion rat model	Lee et al. [99]
Heart and vessel	Alginate	Rat myocardial infraction model	Landa et al. [100]
Cartilage	Collagen	Rabbit articular cartilage	Kubo et al. [101]
	Fibrin	New Zealand white rabbit full thickness cartilage defect	Dai et al. [102]
	Alginate	Rabbit cartilage defect	Ma et al. [103]
Muscle	Collagen	Rabbit muscle	Kin et al. [104]
	Gelatin	Rat muscle	Ju et al. [21]
	Collagen	Rat diaphragm defect	Brouwer et al. [105]
Periodontal tissue	Collagen	Canine periodontal	Nakahara et al. [106]
Skin	Chitosan	Porcine burned skin	Boucard et al. [107]
	Hyaluronic acid-HA	Mouse cutaneous wound model	Wang et al. [108]

**Table 3:** Hybrid biomaterials used for in situ tissue regeneration.

Application Tissue	Biomaterial *	Animal Model	Reference
Bone	Fibrin/PLGA	Rat calvarial bone defect	Chung et al. [109]
	Chitosan/β-sodium glycerol phosphate (CS/GP)	Rat critical size calvarial bone defect	Wu et al. [110]
	PCL-PDA-HAp	Mouse critical size calvarial bone defect	Lee et al. [111]
Cartilage	HA-GelMa	Sheep model	Di Bella et al. [112]
	Collagen-HAp	Human osteochondral defect	Perdisa et al. [113]
	HA-MA/PLGA	Rabbit full thickness cartilage defect	Dai et al. [114]
Blood Vessels	PCL/fibrin	Rat model aorta	Talacua et al. [67]
Heart Valve Leaflets	P4HB/Gelatin	Ovine model pulmonary valve	Capulli et al. [115]
Stomach	Collagen/PGA	Canine stomach	Hori et al. [116]
Spine	PGA/HA	Rabbit disc defect	Abbushi et al. [117]

* ECM—extracellular matrix, PLGA—poly(lactide-co-glycolide), HA—hyaluronic acid, PEUU—polyester urethane urea, PCL—poly(ε-caprolactone), PGA—poly(glycolic acid), PLA—poly(lactic acid), HEMA2—hydroxyethyl methacrylate gelatin methacrylamide (GelMa), HA-GelMa—gelatin methacrylamide (GelMa) and hyaluronic acid methacrylate (HAMA), Hap—hydroxyapatite, P4HB—poly-4-hydroxybutyrate.

**Table 4:** Examples of decellularized extracellular matrix (dECM)-based scaffolds for in situ tissue regeneration

Application Tissue	Biomaterials	Animal Model	Reference
Esophagus	Urinary Bladder Matrix—UBM	Rat Abdominal Esophagus	Keane et al. [130]
Expander/implant breast reconstruction, (a commercial material: Strattice) tissue reconstructive matrix (LifeCell, Branchburg, NJ, USA)	Porcine acellular dermal matrix (PADM)	In humans	Katerinakiet al. [131]
Abdominal wall (a commercial material: Strattice) tissue reconstructive matrix (LifeCell, Branchburg, NJ, USA)	porcine-derived tissue matrix	Primates	Connoret al. [132]
Kidney	PLGA/dECM of porcine kidney tissue	Mouse	Lih et al. [125]
Skin	Porcine subcutaneous adipose tissue	Mouse subcutaneous model	Tan et al. [133]
Hemilarynx	Porcine UBM	Canine model	Kitamura et al. [134]
Heart Valve	PEG/decellularized porcine aortic valve	Rat subdermal model	Dai et al. [127]
	PCL/decellularized porcine aortic valve	Rat subcutaneous model	Zhou et al. [135]

ECM and dECM based materials naturally reflect closely the ECM of the native tissue, but as with natural polymers they face challenges in manufacturing scaffolds with a defined macro-architecture.

**Table 5:** Examples of some bioceramics for in situ tissue regeneration.

Application Tissue	Biomaterials *	Animal Model	Reference
Sinus Mucosa	CP	Rabbit sinus lift model	Trbakovic et al. [155]
Bone	BCP-N	Rabbit mandible critical size defect model	Li et al. [149]
	DNA-loaded nano-calcium phosphate	New Zealand white rabbits, critical size bone defect model	Schlickewei et al. [156]
	PLA/CP	Mouse subcutaneous model	Oliveira et al. [157]
	Calcium-silicate	Rabbit mandibular alveolar bone defect model	Shao et al. [158]
	Alginate/* CSi-Sr_4_ and CaP	New Zealand rabbits, distal femur detect	Fu et al. [159]

* CP (calcium phosphate), BCP-N (nanocrystalline biphasic calcium phosphate), PLA (poly(lactic acid)), CSi-Sr_4_ (strontium-substituted calcium silicate), CaP (beta-tricalcium phosphate).
